# *Illicium verum* anticancer activity against MDA-MB-231 cell line

**DOI:** 10.12669/pjms.40.1.7860

**Published:** 2024

**Authors:** Asra Khan Pahore, Shagufta Khan, Nasim Karim

**Affiliations:** 1 Dr. Asra Khan Pahore, BDS, MPhil. Senior Lecturer, Department of Pharmacology, Altamash Institute of Dental Medicine, Karachi, Pakistan; 2 Dr. Shagufta Khan, MPhil, Ph.D. Assistant Professor, Department of Biological & Biomedical Sciences, Aga Khan University, Karachi, Pakistan; 3 Prof. Dr. Nasim Karim, MBBS, MPhil, Ph.D., Post-Doc. Head Department of Pharmacology, Bahria University Medical & Dental College, Karachi, Pakistan

**Keywords:** Apoptosis, Cell line, Cell survival, *Illicium*, Methanol, Triple negative neoplasms

## Abstract

**Objective:**

To evaluate the anticancer activity of methanolic extract of Illicium verum against triple-negative breast cancer MDA-MB-231 cell line.

**Methods:**

A cell culture experimental study was carried out at Pharmacology department of Bahria University Medical and Dental College (January to June 2021) in collaboration with Aga Khan University, Karachi, Pakistan. Cell viability and proliferation assays were used to quantify dead and alive cells by utilizing a tetrazolium assay and an enzyme immunosorbent plate reader was used to calculate their absorbance. For the apoptosis initiation assay, these cells were dyed with a fluorescent stain and observed for fluorescence and apoptosis. During cell viability testing, various *I. verum* methanolic extract doses (0.125, 0.25, 0.5, 1, 3, 6, 12, and 25µg/ml) were employed to treat MDA-MB-231 cells, while the IC_50_ dose of 2.8µg/ml was used for both the cell proliferation and apoptosis initiation assays.

**Results:**

In the cell viability assay, all *I. verum* methanolic extract doses exhibited a substantial decrease in the viability of MDA-MB-231 cells (less than 0.01 p-value). In cell proliferation assay and apoptosis initiation, the IC_50_ dose of 2.8µg/ml of *I. verum* methanolic extract also exhibited a substantial decrease in cell division (less than 0.01 p-value) and the initiation of apoptosis in MDA-MB-231 cells.

**Conclusion:**

*Illicium verum* methanolic extract have strong anticancer activity against triple-negative breast cancer MDA-MB-231 cell line through cytotoxicity, proliferation reduction, and apoptosis initiation mechanisms.

## INTRODUCTION

Breast cancer is the most widespread female malignancy on a global scale, including Pakistan. Triple-negative breast cancer (TNBC) is a category of molecular classification which relies on receptor expression. It lacks estrogen, progesterone, and HER-2 receptors and represents 12% to 17% of all breast cancer types. TNBC has the worst prognosis, the lowest chance of survival, a high risk of relapse, and metastasizes to the distant organs. Treatment options for TNBC are limited; hormone therapy typically fails due to a lack of receptors.^1^ Chemotherapy is currently the primary treatment option for TNBC; however, because of drug resistance and adverse effects, it has become imperative to find an alternate treatment. Consequently, chemotherapeutic medicines derived from natural sources are being given a high priority.^2^

The medicinal plant contains bioactive compounds and produces biological action.^3^ Numerous bioactive compounds have been identified within these plant species, exhibiting potential anticancer properties, particularly in relation to the treatment of breast cancer. These properties include the ability to induce cell cycle arrest, initiate apoptosis, reduce anti-apoptotic factors, inhibit cell proliferation and metastasis, elevate levels of reactive oxygen species, and enhance antioxidative enzyme activity. Currently, many kinds of chemotherapeutic drugs have been synthesized from these botanical sources, which are also utilized in the treatment and control of breast cancer. Some examples of chemotherapeutic agents used in breast cancer treatment include docetaxel, paclitaxel, etoposide, vinblastine, and vincristine derived from *Taxus brevifolia, Podophyllum peltatum* and *Catharanthus roseus*, respectively.^3^,^4^

* Illicium verum* (*I. verum*), widely known as star anise, is a perennial aromatic plant found primarily in China, Vietnam, and other Asian countries. A phytochemical analysis of dried fruit of *I. verum* revealed the presence of essential oils, monoterpenoids, sesquiterpenoids, phenylpropanoids, lignans, flavonoids, and volatile compounds. These compounds have been attributed to many health benefits, including antimicrobial, antiviral, antithrombin, anti-inflammatory, and anticancer properties.^5^

It has been proven that the trans-anethole component of *I. verum*, inhibits Luminal-A breast cancer cells, as demonstrated by using the MCF-7 cell line. The anticancer activity of *I. verum* has also been documented in human fibrosarcoma and human keratinocyte cell lines.^6^ It has also been proven that the trans-anethole element of star anise inhibits cell growth, causes morphological abnormalities, and initiates apoptosis in human liver and osteosarcoma cell lines.^7^ In another study, the essential oils from the ethanol extract of *I. verum* fruit also showed cytotoxic potential against colon cancer cells through free radical formation, apoptosis initiation, and preventing metastasis.^8^ In a separate study, the polysaccharide component of *I. verum* was shown to inhibit cancer growth in a transplanted S180 (sarcoma 180) mouse model.^9^ In addition, alcohol extract of star anise has shown anti-proliferative and apoptotic action against many cancer cell lines (SW872, SW982, HS 39.T, MCF-7, HT29, and HS 5.T).^10^

In light of the therapeutic significance of *I. verum* extract and the paucity of scientific literature describing its anticancer prospective against multiple cancers, the current study was conducted to evaluate the anticancer activity of methanolic extract of *I. verum* against triple negative breast cancer MDA-MB-231 cell line.

## METHODS

The study was performed at Bahria University Medical and Dental College, Pharmacology Department, in collaboration with Aga Khan University (AKU) (Multidisciplinary and Tissue Culture Lab), Karachi, Pakistan. The chemicals used were Dulbecco’s modified eagle medium (DMEM), phosphate-buffered saline (PBS), trypsin, dimethyl sulfoxide (DMSO), a combination of antibiotic with antimycotic (streptomycin with penicillin and amphotericin-B), triton-x, 3-(4,5-dimethylthiazol-2-yl)-2,5-diphenyl-2H-tetrazolium bromide (MTT), fetal bovine serum (FBS), 4´, 6-diamidino-2-phenylindole fluorescent nuclear dye (DAPI), paraformaldehyde, methanol, and ethanol.

### Ethical Approval

This cell culture experimental study was a part of MPhil thesis research, carried out for six months (January to June 2021) after approval of Ethical Research Committee (BUMDC-ERC letter #11/2021, dated: 15-01-21).

For extract preparation, five kilograms of *I. verum* dried fruit was acquired from a marketplace. A voucher copy (IV-F-27-12-120) was submitted and *I. verum* dried fruit was immersed in five liters of solvent (aqueous methanol 30:70) for three days at room temperature in MDL, AKU. The muslin fabric and Whatman grade-1 filter paper were used to get the first filtrate. The fruit residue was immersed again in a similar ratio of aqueous methanol for three days at room temperature each to obtain the second and third filtrates. Later all three filtrates were mixed and a rotary evaporator (Model Buchi: R210, Switzerland) was used to evaporate the remaining solvent to get the final *I. verum* methanolic extract. This extract was kept at 4ºC and used for the assays.^11^,^12^

For the growth of MDA-MB-231 cells (a TNBC cell line by ATCC, Manassas, VA, USA), it was cultured in DMEM, which contains a mixture of FBS (10%) and antibiotic-antimycotic (1%). In cell viability assay, 1×10^4^ MDA-MB-231 cells were placed in each well of 96-well culture plate with 100µl of DMEM. After 24hrs of incubation, *I. verum* methanolic extract was added to the cells in various doses (0.125, 0.25, 0.5, 1, 3, 6, 12, 25µg/ml) and incubated for 48hrs. Following incubation, the previous media was changed with new media containing MTT dye 0.5mg/mL and incubated at 37ºC in a 5% carbon dioxide (CO2) humidified air for four hours. To determine cell viability, the formazan crystals produced by living cells were dissolved in DMSO, and an enzyme-linked immunosorbent assay plate reader (ELISA Model Bio-Rad: iMark No.168-1135, USA) with a wavelength of 550nm was used to measure the absorbance of these crystals. Using the following formula, the percent of dead and alive cells was determined:^13^

Percent cell viability = {(tested – blank) / (control – blank)} × 100

In cell proliferation assay, 1×10^3^ MDA-MB-231 cells were placed in each well of 96-well culture plate with 100µl of DMEM. After 24hrs of incubation, *I. verum* methanolic extract IC_50_ dose 2.8µg/ml (obtained from the previous assay) was given for 24 to 72hrs. Using the above-mentioned formula, the surviving cells were counted after specified time intervals. Later, these cells were used to plot the cell growth curve over the time of 24 to 72hrs.^13^

For apoptosis initiation assay, the DAPI staining test was conducted identically to the previous ones, with few minor modifications.^14^ In a 96-well culture plate, 1×10^4^ MDA-MB-231 cells were placed in each well with 100µl of DMEM and incubated at 37ºC in a 5% CO2 humidified air for 4hrs. Following incubation, *I. verum* methanolic extract IC_50_ dose 2.8µg/ml was given to these cells for 48hrs. After removing the medium containing the extract, the cells were cleaned with PBS and fixed for 10 minutes with paraformaldehyde (4%). Later, 0.5% triton x-100 and 4% paraformaldehyde (buffer) were used to make the cells permeable, and they were stained for 1hr with 50µl DAPI with 1µg/ml dose. After the allotted time, the fluorescence intensity and apoptosis of the stained cells were evaluated, and pictures were taken. A fluorescent microscope was used to quantify these cells. Using the following formula, the percent of apoptotic cells was calculated:^15^

Percent apoptotic cells = (cells of control – cells with apoptosis) × 100

### Statistical analysis

The data was represented as the mean and standard error mean and assessed with GraphPad Prism four software using one-way analysis of variance (ANOVA) and Dunnett’s comparison tests. The ANOVA test indicates significant findings relative to the control group. While the Dunnett test indicates a significant variation in the effects of each dose and time period on the cell. The results were deemed statistically significant when the p-value was below 0.05.

## RESULTS

**Fig.1:**
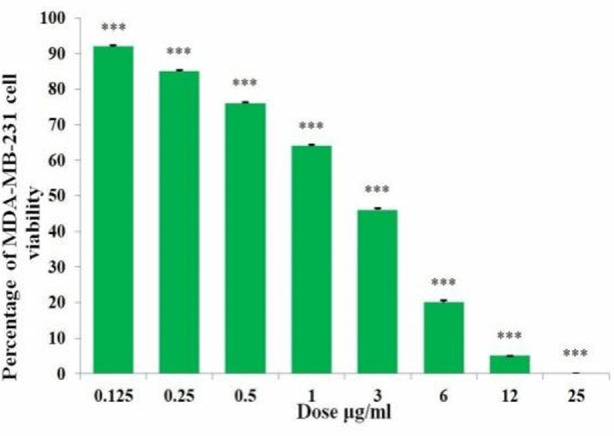
The effect of *I. verum* methanolic extract doses on the MDA-MB-231 cell viability.

After 48hrs of incubation, *I. verum* methanolic extract significantly reduced the viability of MDA-MB-231 cells in relation to the dose (0.125 – 25µg/ml). As shown in Fig.1 at the minimum dose of 0.125µg/ml, the extract displayed 91.7% cell viability, whereas, at the maximum dose of 25µg/ml, the extract displayed complete inhibition of cell viability (0%). Each value is the mean and SEM of three measurements performed in triplicate.

After 48hrs of incubation, *I. verum* methanolic extract significantly increased the death of MDA-MB-231 cells in relation to the dose (0.125 – 25µg/ml). As shown in Table-I, the extract caused 8.3% cell death at 0.125µg/ml and reached maximum cell death (100%) at 25µg/ml. Each value is the mean and SEM of three measurements performed in triplicate.

**Table-I:** The effect of *I. verum* methanolic extract doses on MDA-MB-231 cell death.

Dose (μg/ml)	0.125	0.25	0.5	1	3	6	12	25
Mean	8.33	15.33	24.33	35.66	54.00	80.00	95.00	100.00
Standard deviation	0.57	0.57	0.57	0.57	1.00	1.00	0.00	0.00
Standard error	0.33	0.33	0.33	0.33	0.57	0.57	0.00	0.00
p-value	<0.01	<0.01	<0.01	<0.01	<0.01	<0.01	<0.01	<0.01

**Fig.2:**
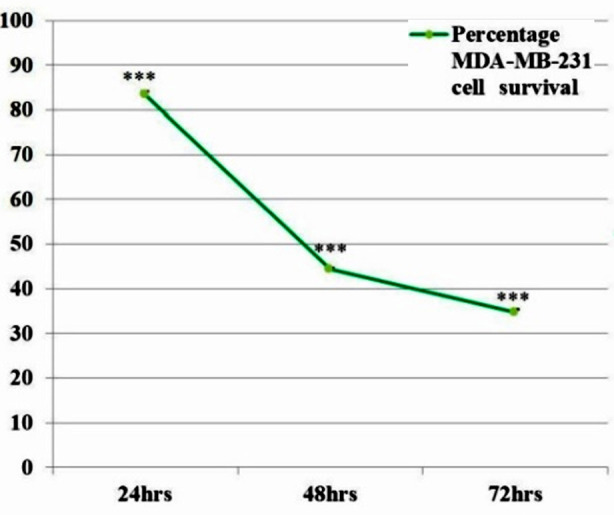
The effect of *I. verum* methanolic extract IC_50_ (2.8µg/ml) dose on MDA-MB-231 cell survival during 24 to 72hrs.

When the IC_50_ dose of 2.8µg/ml of *I. verum* methanolic extract was given to treat MDA-MB-231 cells for 24 to 72hrs, the extract significantly reduced the growth of these cells relative to the respective control (untreated cell growth). As illustrated in Fig.2 MDA-MB-231 cell growth was 83.3%, 44.3%, and 34.7% respectively. Each value is the mean and SEM of three measurements performed in triplicate.

When the IC_50_ dose of *I. verum* methanolic extract was given to treat MDA-MB-231 cells for 24 to 72hrs, the extract significantly increased the death of these cells relative to the respective control (untreated cell growth). As shown in Table-II, MDA-MB-231 cell death was 16.7%, 55.7%, and 65.3% respectively. Each value is the mean and SEM of three measurements performed in triplicate.

**Table II:** The effect of I. verum methanolic extract IC_50_ (2.8µg/ml) dose on MDA-MB-231 cell death during 24 to 72hrs.

Dose (μg/ml)	2.8	2.8	2.8
Hours	24	48	72
Mean	16.66	55.66	65.33
Standard deviation	1.52	0.57	1.15
Standard error	0.88	0.33	0.66
p-value	<0.01	<0.01	<0.01

The DAPI fluorescence microscopy revealed that treatment of IC_50_ dose of *I. verum* methanolic extract with the MDA-MB-231 cells for 48hrs caused condensation and segmentation of the nuclei.

## DISCUSSION

The findings of our study indicated that the *I. verum* methanolic extract decreased the viability of MDA-MB-231 (TNBC) cells in relation to dose, with an IC_50_ dose of 2.8 ± 0.6µg/ml. Providing evidence of its anticancer activity against triple-negative breast cancer cells (Table-I). Similarly, studies of methanolic extracts of *Scurrula ferruginea*, *Withania somnifera*, *Christia vespertilionis*, and *Asparagus racemosus* plants were reported to possess anticancer activity with an IC_50_ dose of 19µg/ml, 30µg/ml, 37µg/ml, and 91µg/ml via cell viability assay, respectively.^16^-^19^

Following the remarkable cytotoxicity results of *I. verum* methanolic extract, its anti-proliferative potential against MDA-MB-231 cells was also assessed. In the present study, an IC_50_ dose of 2.8µg/ml of the same extract inhibited the growth (proliferation) of MDA-MB-231 cells at 24 to 72hrs, respectively (Table-II). Similar studies have been done to evaluate the anti-proliferative potential of plants against MDA-MB-231 cell lines. Notably, it has been documented that the methanolic extracts of *Berberis hispanica*, *Solanum schimperianum*, and *Abrus precatorius* inhibited MDA-MB-231 cell proliferation by 50% after 24 to 72hrs of incubation at a dose of 16.5 ± 0.6µg/ml, 7 ± 0.25µg/ml, and 26.4 ± 5.46µg/ml, respectively.^20^-^22^

Apoptosis (programmed cell death) is a process in which cells self-destruct when triggered by the appropriate stimuli. Typically, the cells are maintained and regulated by a constant balance between cell division and apoptosis. Any disruption of this equilibrium leads to a change in homeostasis and the eventual development of malignant cells. Thus, apoptosis and cell cycle arrest are one of the key aspects of breast cancer prevention and suppression. There is considerable interest in the research of new apoptosis-initiating drugs derived from natural sources with higher potency, specificity, and less toxic side effects.^23^ In our study, *I. verum* methanolic extract demonstrated the initiation of apoptosis in MDA-MB-231 cells at an IC_50_ dose of 2.8μg/ml, confirming that its cytotoxic effect is attributed to apoptosis. Previous studies using the DAPI staining assay have similarly shown that extracts from *Petasites hybridus*, *Artemisia nilagirica*, and *Zanthoxylum armatum* also initiate apoptosis in these cells, thereby exhibiting anticancer activity.^24^-^26^ Consequently, these findings are compatible with the results of previously described experiments suggesting that the *I. verum* methanolic extract is efficient against MDA-MB-231 cancer cells.

The increased rate of morbidity and mortality in triple-negative breast cancer can be attributed to ineffective treatment options. Given the limited success of conventional treatment choices, the goal of this research was to explore a new therapy approach for TNBC, as indicated by the MDA-MD-231 cell line. While many prior studies have focused on the curative effects of *I. verum*, very limited research has been conducted on its anticancer effects. This study stands out as no documented study has been found globally or locally upon a literature search of ten years from 2013-2023 using the search engines Google Scholar and PubMed on our study topic. A methanolic extract of *I. verum* dried fruit is speculated as an alternative treatment option for triple-negative breast cancer based on our study findings. Therefore, our results are generating new opportunities for future researchers to undertake clinical trials utilizing *I. verum* methanolic extract of dried fruit to further evaluate its potential in triple-negative breast cancer.

### Limitations of the study

Due to financial constraints and the unavailability of different breast cancer cell lines in Pakistan during the study period, only MDA-MB-231 cell line was utilized.

## CONCLUSION

*I. verum* methanolic extract has exhibited a strong anticancer activity against triple-negative breast cancer MDA-MB-231 cells through cytotoxicity, proliferation inhibition and reduction, and apoptosis initiation mechanisms.

### Author’s Contribution:

**AKP:** Primary investigator, responsible for collecting data, literature review, and manuscript preparation.

**SK:** Co-supervisor of the research, performed data analysis and interpretation.

**NK:** Supervisor of the research, planned and designed the study and critically edited the final article.

**AKP, SK and NK:** All authors are responsible for the accuracy of the study.
